# Summary adherence estimates do not portray the true incongruity between drug intake, nurse documentation and physicians’ orders

**DOI:** 10.1186/1471-2369-15-170

**Published:** 2014-10-23

**Authors:** Inbal Cohen-Glickman, Yosef S Haviv, Matan J Cohen

**Affiliations:** School of Medicine and School of Pharmacy, Hadassah-Hebrew University Medical Center, Jerusalem, Israel; Dialysis unit, Nephrology and Hypertension services, Hadassah-Hebrew University Medical Center, POB 12000, En Kerem campus, Jerusalem, 91120 Israel; Department of Medicine and the Center for Quality and Clinical Safety, Hadassah-Hebrew University Medical Center, Jerusalem, Israel

**Keywords:** Hemodialysis, Adherence, Medications, Pharmacy, Physician orders

## Abstract

**Background:**

Hemodialysis patients (HD) need to adhere to a complex medication regimen. Because their daily pill burden is one of the highest reported, poor compliance is a major cause of therapeutic failure. The primary aim of this study was to define patterns of medication prescription, intake and documentation among HD patients.

**Methods:**

HD patients treated between 2007 and 2009 and assigned to the largest health service provider in Israel were randomly selected. Drug practices were abstracted from their records and compared to electronic pharmacy data. The discrepancy between drug intake reports and the actual purchase was measured to estimate adherence. Drug purchase, intake report and physician order were plotted in complementing diagrams to appreciate consistency and discrepancy patterns.

**Results:**

The study included full analysis of 75 patients. The mean overall drug adherence was 56.7% (95% CI 53.6-59.9%), varying among drug families and over time. Often, there was a systematic disengagement between the nurses’ documentation and the actual patient purchase. Specifically, we observed either different quantities of medication use, improper documentation of a non-purchased drug, drug purchase without nurse documentation and futile physician attempts to modify prescriptions of unpurchased medication. We found a high rate of physician order turbulence for active vitamin D and calcium.

**Conclusions:**

Drug prescription, documentation and adherence are incongruent and their mismatches are diverse. Summary estimates do not divulge the extent of these disparities. These system-wide communication failures compromise patient care. Strategies to promote system reconciliation and reasonable medication prescription are in need.

**Electronic supplementary material:**

The online version of this article (doi:10.1186/1471-2369-15-170) contains supplementary material, which is available to authorized users.

## Background

Hemodialysis (HD) patients are prescribed numerous oral medications [1], averaging 17 to 25 doses per day [2, 3]. This high medication burden results from the complexity of end stage renal disease and its comorbidities [4]. Previously, patterns of medication use in USRDS HD patients were reported using descriptive analyses where the rate of non-adherence to medications in HD patients ranged from 15.4% to 50.2% [4–7], culminating to 74% when phosphate binders were considered [8]. Because non-compliance with prescribed treatment in the general population, especially medications, increases both the cost of health-care and the likelihood of hospital admissions [8], the World Health Organization has set action-plans to improve general patient adherence to long-term therapies [1, 9].

Integrated care for HD patients is a complex treatment mandating four different types of adherence: medication adherence, dietary restrictions, regular attendance of dialysis sessions and keeping up with scheduled appointments. While non-adherence to HD sessions, in the form of missing or shortening sessions, is associated with higher mortality [6, 9], it is assumed, but not proven, that non-adherence to medications in general, and to specific drug groups in particular, may also be associated with unfavorable outcomes in HD patients.

The terminology, definitions, and methods used to determine medication adherence vary and include medication possession ratio, discontinuation and continuation rates, switching rates, treatment gaps and turbulence [10, 11]. While current methods to assess adherence often rely on patient self-report [2, 12, 13], thereby limiting cross-study comparisons, only a few studies have systematically compared the properties of different adherence measures [14, 15]. In this study, we sought to critically measure medication adherence in HD patients over a long period and to evaluate drug-related practices by nurses and nephrologists. We compiled all documented medication prescription, drug purchase and reported intake into a single repository using pharmacy electronic medical records and review of HD units’ medical files.

## Methods

### Patients

This study was exempted from the requirement to obtained patient consent for inclusion in the study. A General IRB approval from the Hadassah Medical Organization Helsinki committee was obtained for comprehensive retrospective, non-interventional, epidemiology research in dialysis patients. This approval was given as these studies do not compromise patient autonomy, do not influence the care patients receive. In many cases, due to the historical-retrospective nature, the study includes patients who are already deceased. Access to patient database and use of patient data are allowed, so long as research records and report are anonymous.

The study participants were treated in seven outpatient HD units directed by nephrologists affiliated with the Hadassah-Hebrew University Medical Center, Jerusalem, Israel. The majority (~65%) of our patients are insured by Clalit Health Services (CHS), the largest health maintenance organization in Israel, which provided electronic pharmacy drug dispensing data for its HD patients. There were no minors in the study.

Of all chronic prevalent HD patients insured by CHS and treated between July 1^st^, 2007 and July 1^st^, 2009, half of patients were randomly selected using an arbitrary digit of their ID number. Exclusion criteria included acute kidney injury, dialysis start date before January 1st, 2006 and age <18 years. Censorship criteria were: kidney transplantation, death, loss to follow-up and end of follow-up period.

### Medications

The study included 13 different groups of oral drugs commonly prescribed to HD patients (Table 1). In the current study we documented oral drug intake that could be further grouped into three major drug groups: drugs for atherosclerosis (aspirin, nitrates, statins), drugs for heart failure/arrhythmia/fluid overload/blood pressure (ACE inhibitors/ARB, beta blockers, calcium channel blockers, doxazosin, furosemide, anti-arrhythmics), and mineral bone disease agents (calcium carbonate, alfacalcidol). In addition, data were collected for oral hypoglycemics and anti-depressants. At the time of the study only a minority of patients were prescribed non calcium-based phosphate binders, thereby resulting in exclusion of this class. Oral anticoagulants were excluded due to overlap of prescription with general practitioners and weekly dose changes.

**Table 1:** **Medications groups**

	Frequency (No)	Percent (%)
**Antidepressants**	10	2.2
Mirtazapine, paroxetine, escitalopram, amitryptiline, fluvoxamine, escitalopram		
**Doxazosin**	13	2.9
**Anti-arrhythmics**	13	2.9
Amiodarone, flecainide, propafenone		
**Oral hypoglycemics**	18	4.0
Glibencamide, repaglinide, acrabose, metformin		
**Nitrates**	20	4.5
Glyceryl trinitrate,Isosorbide mononitrate,Isosorbide dinitrate		
ACE inhibitors and ARB’s*	27	6.0
Captopril, ramipril, enalapril, losartan, cilazapril, valsartan		
**Furosemide**	32	7.1
**Calcium Chanel Blockers**	33	7.3
Felodipine,amlodipine,nifedipine, verapamil,lercandipine		
**Beta Blockers**	46	10.2
Metoprolol, Atenolol, Carvedilol, Bisoprolol		
**Statins**	49	10.9
Simvastatin, Pravastatin, Atorvastatin		
**Aspirin**	53	11.8
**Calcium Carbonate**	67	14.9
**Alfacalcidol**	68	15.1
**Total**	449	100.0

*Includes 4 cases of combined ARB’s and Hydrochlorothiazide in one pill.

### Medication practice in HD units

Patient drug intake is commonly recorded in HD units on a monthly basis by the nursing staff via direct patient questioning. Next, during the physician’s visit, the medication list serves as the basis for new orders or dose adjustments after obtaining new clinical and laboratory data. We used the nurses’ manual drug form presented to the physicians, to document reported medication intake. We next obtained information about drugs actually purchased each month from the CHS electronic database. Because HD patients are exempt from payment when purchasing most disease-related drugs through CHS pharmacies, drug purchase elsewhere is unlikely. We recorded for each drug: a) the amount of pills actually purchased every month, as recorded in the electronic pharmacy database; b) the amount of medication supposedly consumed, recorded in the nurse reports; c) physicians’ recorded medication decisions, i.e. change of regimen/dosage (stop, increase or decrease dose, or start a new medication).

### Electronic pharmacy database

In CHS pharmacies medications can be purchased in three-month intervals. To correct for potential adherence underestimation, we standardized episodes of excess medication purchase intake into one month purchase. For example, when a patient purchased pills sufficient for more than one month with no purchase on the following two months, we allowed the excess amount of pills to be normalized per month. We did not perform correction for excess medication purchase of calcium carbonate and activated vitamin D analogs (alfacalcidol) since there were frequent changes in doctors’ orders for these drugs, often on a monthly basis. Drugs from the same class were considered interchangeable for the purpose of adherence estimation. We documented switches between drugs of the same family in the doctor’s orders column.

### Nurse reports

The nurses’ reports served as the basis to calculate the expected number of medications consumed monthly. Textual comments could not be quantified and were not included in our estimation of adherence. This potential bias might skew our analyses towards overestimation of adherence and under-estimation of non-adherence. However, nurses rarely used text to document the amount of pills taken (“as needed”, “sometimes forgets” etc.). We also corrected for any discrepancy which could arise from purchasing packages of 28 pills when nurse documented 30 pills purchases. When a nurse documented a range of doses instead of a fixed quantity, we used the average amount for calculations. For example, if a patient was documented to use 60–90 pills monthly, we considered it as 75 pills.

### Calculation of adherence

To measure adherence we assumed that all nurse-documented drug information was available to the nephrologist from the medical file. In cases where there were no new orders, the medication lists were regarded as approved by the nephrologists. In the following month we checked whether the patient purchased the drugs again using methodology as described above. We defined adherence or non-adherence as a dichotomous variable. For each drug we compared the number of months the drug was actually purchased to the number of months of declared drug intake as reported by the patients to the nurses. This ratio was defined as patient adherence (%). To avoid non-adherence designation due to ignorance of doctors' orders, when there was no nurse documentation, these months were omitted from calculation of adherence, thereby possibly causing to over-estimation of adherence.

In addition to measuring adherence, we recorded instances designated as over-adherence, i.e. use of the drug without doctors’ orders. When an order to stop the drug was recorded, yet in the following months the medication was nevertheless purchased, we considered the patient over-adherent.

Patient purchases of medications in the absence of clearly identified orders to stop these medications were excluded from definition of over-adherence. The percentage of over-adherence was calculated as the ratio between the number of months a patient purchased the medication and the number of months since the order to stop the drug, extending either until the end of data collection or until a new order was recorded. For example, if an order to stop a medication was given and followed by 12 months of follow-up, in all of which the patient continued to purchase the drug and did not inform the nurse and nephrologists, the over-adherence was 100%.

### Individual patterns of drug practices

**Figure 1:**
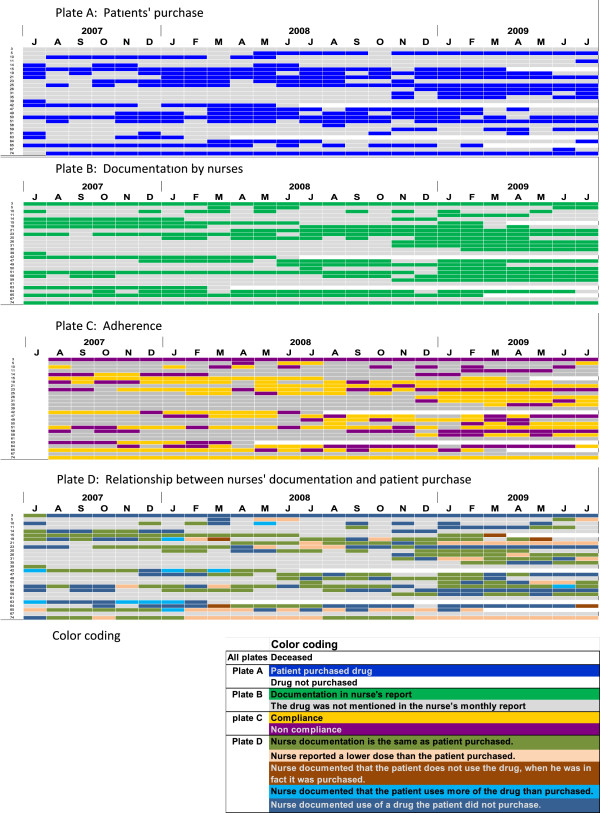
**Medication purchase, reporting and adherence patterns of ACE inhibitors and ARB’s, in the form of color medication plates.** The plates reflect patient purchase patterns **(A)**, nurse reported intake **(B)**, patient adherence calculations **(C)** and purchase and dose consistencies **(D)**. Each row represents a patient and each column represents a month, rows are numbered from 1 to 75 and each represents a patient in our study. **E** depicts color coding of the medication plates.

In addition to calculating the adherence rate, we found during data collection that the patterns of medication purchase and reported intake were inconsistent to the extent that merely presenting descriptive statistics would have underscored the patchiness of the phenomenon. We therefore sought to present the raw data itself to allow a better appreciation of what we perceived as therapeutic pandemonium (Figure 1 and Additional file 1: Figures S2-S12). In these diagrams, drug intake behavior of individual patients is depicted in each row and each column represents a calendar month. These comprehensive plates reflect patient purchase patterns (designated A), nurse reported intake (designated B), patient adherences (designated C) and purchase and dose consistencies/discrepancies (designated D) in the form of color medication plates. Each plate depicts one drug or a family of drugs. The rows are numbered from 1 to 75 and each represents a patient in our study. Each patient is consistently represented by the same number. In all plates, white bars reflect patient death.

## Results

### File allocation and patient survival

Of the 110 eligible HD patients, manual medical records of 28 patients (25.4%) could not be located in the archives of the dialysis units and in 7 (6.4%) files, the medical record lacked substantial documents. We therefore analyzed 75 (68%) patient files with a mean patient follow up time of 23.62 (SD 3.8) months of the planned 25 months.

The mortality rate was lower (17.3%) in the analyzed group compared to the initial group (29.1%) (Table 2). Because both drug purchase and intake require either intact cognitive function or assistance, it is of note that 90% of analyzed patients had intact cognition and 61.3% had access to a caregiver if needed.

**Table 2:** **Baseline characteristics of the study participants**

		Patients included in analysis N = 75		Patients randomly chosen for research N = 110	
**Age at start of research, Mean/SD**		64.55	15.94	65.27	15.35
**Died during follow/up, N/%**		13	17.3%	32	29.1%
**Sex female, N/%**		31	41.3%	0.47	42.7%
**Transplanted before dialysis, N/%**		4	5.3%	5	4.5%
**Current smokers, N/total available N/%**		10/63	15.9%	12/72	16.7%
**Residence during study period,** N/total available N*/%					
	Private residence	64/70	91.4%	72/79	91.1%
	assisted living/nursing home	6/70	8.6%	7/79	8.9%
**Primary care taker,**					
N/total available N*/%					
	Partner	19/57	33.3%	22/79	27.8%
	Children	10/57	17.5%	11/79	13.9%
	hired care	6/57	10.5%	10/79	12.7%
	Other	5/57	8.8%	5/79	6.3%
	none/not mentioned	17/57	29.8%	18/79	22.8%
**Mental and cognitive disability**					
N/total available N*/%					
	No disability	63/70	90.0%	71/78	91.0%
	Dementia	2/70	2.9%	2/78	2.6%
	Other cognitive/mental disability	5/70	7.1%	5/78	6.4%
**Mobility**					
N/total available N*/%					
	Mobile	40/61	65.6%	43/67	64.2%
	Walking aid	7/61	11.5%	8/67	11.9%
	Walker	6/61	9.8%	7/67	10.4%
	Wheel chair	8/61	13.1%	9/67	13.4%

**Figure 2:**
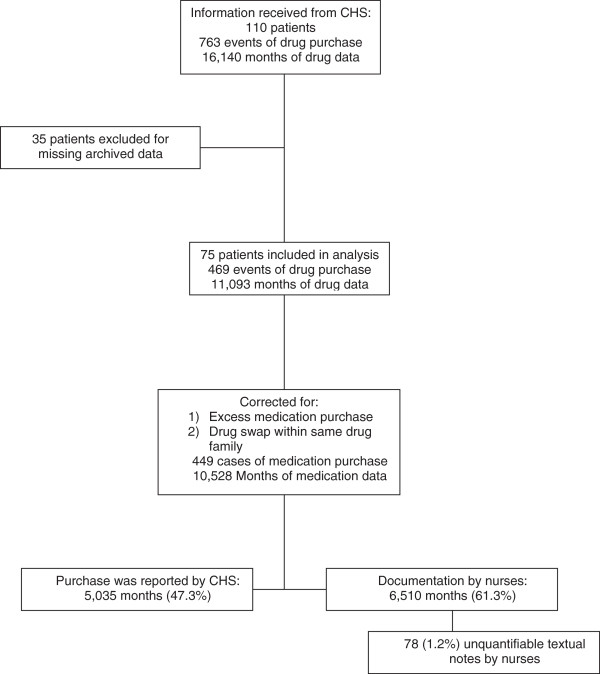
**Flow chart showing patients and data inclusion in the study.** *CHS-Clalit health services.

*Since the different Dialysis units affiliated with our institute do not document patient information in an identical manner, not all information presented was availed for all patients. See text and Figure 2 for exclusion criteria.

### Clinical indications for drug purchase in HD patients

The frequency of drug use by HD patients is described in Table 1. There were three major indications for medications in our HD patients; a. Medications related to mineral bone disease comprising 30% of all drugs. b. 36.4% of drugs purchased were indicated to treat heart failure, hypertension or arrhythmia (beta or calcium blockers, ACE inhibitors or ARB, furosemide, doxazosin and anti-arrhythmics). c. drugs for atherosclerosis (nitrates, aspirin and statins) comprising 27.2%. Only 4% of oral drugs purchased were indicated for diabetes despite a 10-fold higher rate of diabetes.

### Characteristics of drug purchase vs. nurse reports

The electronic CHS purchase database included a total of 763 medications purchased and 16,410 months of medication data for the entire study population of 110 patients. The 75 analyzed patients had CHS records documenting 469/763 (61.5%) medication purchases during a total of 11,093 months of medication data. Drugs were purchased only during 4,643 months (41.9%) of 11,093 months and nurse report of drug intake was documented in 6,497/11,093 months (58.6%). After correcting for excess medication purchase and accounting for medication switches within the same class (see methods), there were 449 drugs used over a period of 10,628 months of medication data. Drugs were purchased during 5,035/10,628 (47.3%) months and nurses drug reports were documented in 6,510/10,628 months (61.3%).

In 78/6,510 (1%) of these months there were textual comments in the nurse reports which could not be quantified (for example “takes when needed”) (Figure 2).

**Figure 3:**
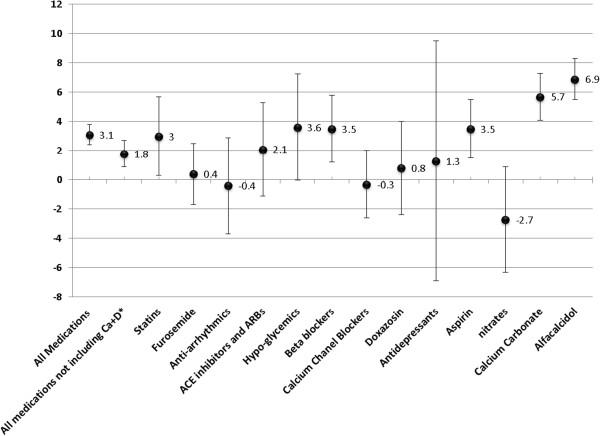
**Differences between nurse reports and actual drug purchase.** Error bars indicate 95% confidence interval. A positive value indicates the nurses documented medication use than actually purchased, A negative value indicate more drugs purchased than reported by nurses. *Calcium carbonate and alfacalcidol.

During a follow up period of 25 months, the average (SD) time span of drug purchase was 11.2 (7.8) months and the nurse documented patient intake for each drug was on average (SD) 14.5 (8.0) months (Table 2). Thus, for most medications, documentation by the nurse exceeded patient purchases (Figure 3). This difference was primarily evident for alfacalcidol and calcium carbonate but could also be observed for aspirin, statins and beta-blockers. For furosemide, calcium channel blockers and antiarrhythmics, nitrates and doxazosin, the rates of patient purchase and nurse reporting were similar. The mean patient-nurse concordant months (months in which the nurse reported exactly what the patient purchased) was 5.7 months (95% CI 5.2-6.4), which comprised 50.9% of the purchase months and 39.3% of the reported intake months were matched. After excluding calcium carbonate and alfacalcidol, the mean number of concordant months was 7.1 (95% CI 6.4-7.8). For example, patient # 2 did not purchase beta blockers at all throughout the entire follow-up period, while it was “documented” by the nurse (Additional file 1: Figures S2-A to S2-D); patient # 54 purchased a higher dose of statins than documented by the nurse for most of our study period (Additional file 1: Figure S3-D). Thus, nurses’ reports and actual patient purchase were often incompatible, sometimes over long periods.

### General non-adherence and over-adherence

**Figure 4:**
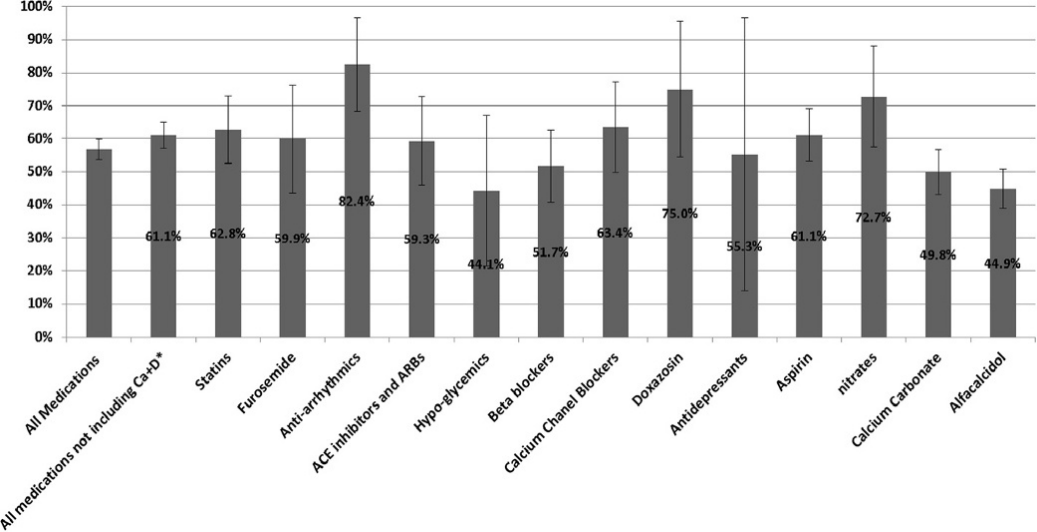
**Variation of adherence rates among the different drug groups.** *Calcium carbonate and alfacalcidol.

On average, the overall adherence for all drugs was 56.7% (95% CI 53.6-59.9%) (Figure 4). The adherence to anti-arrhythmics was high and to calcium carbonate, alfacalcidol and oral hypoglycemic agents was low. Over-adherence occurred in 23.6% (95% CI 16.3-30.9%) of the months following an order to stop medications.

Patterns of individual drug intake, as shown in the medication plates (Figure 1 and Additional file 1: Figures S2-S12), indicate that in several cases the medications had not been purchased for long periods, despite the nurse reporting medication use. We also noted cases where a medication was purchased without any nurse reports. In Figure 1 and Additional file 1: Figures S2-S12, plates D present regimen comparison between patient purchase and nurse reporting. In some of the figures we demonstrate instances where a drug was purchased at different doses than those documented by the nurse.

As explained in the methods section, plates D do not take into account cases where the nurse report for that month was empty. Over-adherence at several levels was noted for furosemide: we documented only 15 doctors’ orders for furosemide for 32 patients during 469 patient-months, much less than would be expected in view of the frequent changes in medication purchase which were also under-documented by the nurses (Additional file 1: Figure S7).

### Turbulence in physician orders

**Figure 5:**
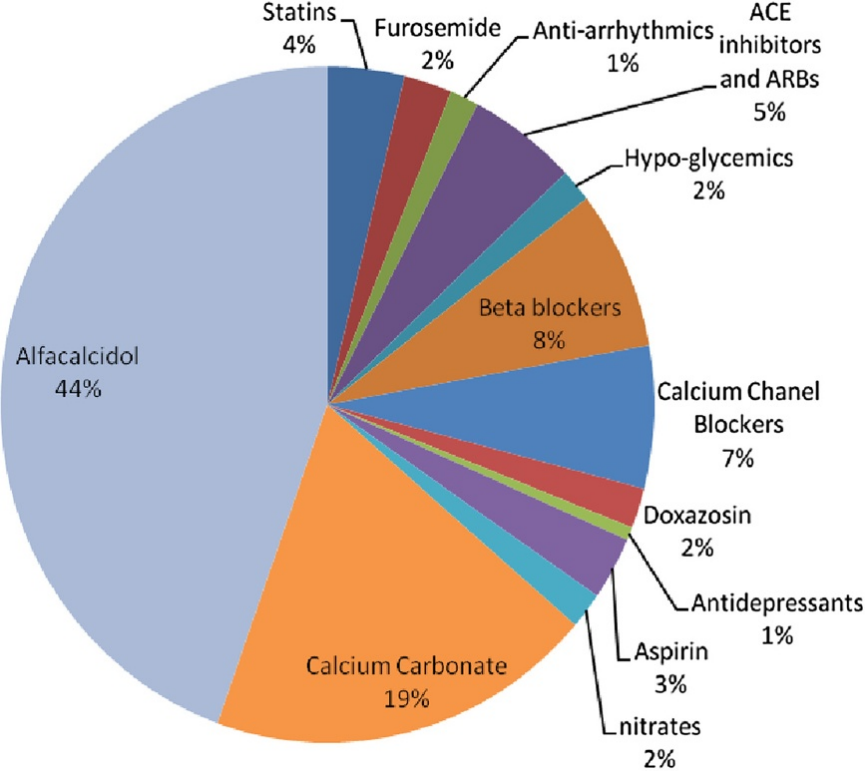
**Doctors’ orders by drug type.** The proportion of orders regarding each drug family, of all orders recorded in our study.

We documented 632 nephrologists’ orders for changes in medication regimen (Table 3, Figure 5). Drug turbulence is a qualitative measure defined as a high rate of change in physician orders for a given drug. While calcium carbonate and Alfacalcidol together comprised 33.2% of the drugs in our study, 63.6% of physicians’ orders involved these two drugs, thereby positioning this class of mineral bone disease drugs as the major drug class occupying doctors’ time (Table 3). For comparison, aspirin, prescribed for long periods at a fixed dose, manifested a very low turbulence rate. However, to more carefully focus on turbulence associated with non-adherence, we hypothesized that of all possible physician orders (i.e. start a new drug or stop, reduce or increase dose, or switch to another drug within the same group), an order to increase drug dose would better reflect non-adherence. Alfacalcidol uniquely required more orders to increase drug dose than decrease dose (142/281 of alfacalcidol orders vs. 46/121 for calcium carbonate and 38/230 for all other medications, Table 3), thereby reflecting a very high level of non-adherence for this drug. This finding is further corroborated by the low (44.9%) calculated adherence rate for alfacalcidol (Figure 4) and by its low purchase rate relative to the nurses reports (Figure 3).

**Table 3:** **Doctors' orders in HD units**

	All medications not including Ca + D	Calcium carbonate	Alfacalcidol	All medications
**Start treatment**	**71**	**17**	**36**	**124**
**Increase dose**	**38**	**46**	**142**	**227**
**Reduce dose**	**32**	**36**	**57**	**125**
**Stop the treatment**	**66**	**22**	**46**	**134**
**Switch to different drug of the same family**	**23**			**23**
**Total**	**230**	**121**	**281**	**632**

### Detailed patterns of drug purchase and adherence

Medication Plates shown in Figure 1 and the Additional file 1: Figures S2-S12, indicate the drug purchase patterns in the form of color medication plates as described in methods. Typical patterns of drug purchase, nurse report and adherence for ACE inhibitors and ARBs are presented in Figure 1 - plate A and plate B show medication purchase in blue and documentation by the nurse in green, respectively. Lack of purchase or documentation is marked in grey. Mismatch between 5A and 5B depicts pharmacy purchase vs. nurse drug documentation.

The two plates differ substantially with regard to several months when patients did not purchase the drug but the nurses did report usage, and vice versa. While the descriptive data (Figure 3) indicate a small difference between patient purchase and nurse reports (10.3 vs. 12.4 months), the plates’ qualitative data in Figure 1 indicate a far more complex discrepancy. For example, in the case of patient # 3 (first row) although the nurse documented regular use of the drug, the patient did not purchase it at all. In the case of patient # 64, on July 2008 the patient stopped using the drug altogether, but the nurse kept on reporting it is being used.

Figure 1 plate C contrasts and completes the adherence rates presented in Table 2, as it illustrates that the crude calculated rates do not depict the hectic adherence patterns. Figure 1C shows adherence with doctors’ orders as previously defined; in the month following nurse documentation, the patient was expected to take the medication as previously reported, unless instructed otherwise following a physician’s order. Patient # 3 is considered non-adherent throughout the whole study period since the drug had never been purchased although the nurse kept reporting it was taken. Patient # 75 is considered adherent throughout the entire study, since the drug was purchased and documented by the nurse throughout the entire study period. The example of patient # 5 illustrates drug purchase almost consecutively from July 2008, but because the nurse hardly reported drug use, adherence of this patient could not be determined. Finally, patient # 10 purchased the drug on nine months of the study and the nurse reported drug use during nine months of the study, but because these months rarely coincided, this patient was considered adherent during only 3 of the 9 months reported by the nurse (33%).

Figure 1 plate D incorporates only cases where the nurse reported drug intake and demonstrate dosage inaccuracies. In Figure 1D, patient # 74 purchased a higher dose of the drug compared to the nurse documentation (colored tan) for a long consecutive period of time. There were separate events (marked in brown in Figure 1D) where the nurse specifically reported no drug use, despite documented concomitant drug purchase. These patterns differ from months when the nurse disregarded the drug altogether, while the drug was purchased (marked grey in Figure 1B).

Similarly to the pattern observed for ACE inhibitors and ARB’s, the online supplementary figures show plates of beta blockers, statins, aspirin and calcium channel blockers portraying discrepancies between patient purchase (plates labeled A) and nurse documentation (plates labeled B). Plates labeled C, which represent adherence to physician recommendations, indicate only a few patients who were consistently adherent or non-adherent (for example for statins, Additional file 1: Figure S3 plate C, patient # 20 was consistently adherent while patient # 73 was consistently non-adherent). Most patients using statins were adherent yet demonstrated sporadic non-adherence periods. Taken together, the above data indicate chaotic drug intake patterns.

### Adherence is higher for symptom-relieving medications

The HD patients studied were more adherent to nitrates, anti-arrhythmics and doxazosin (Additional file 1: Figures S3-S5- plates C). These medications also manifested less frequent discrepancies between nurse reporting and patient purchase (Additional file 1: Figures S4-S6- plates A, B, D). This finding could also be demonstrated in Figure 3. Furosemide (Additional file 1: Figures) appears to have a unique profile as evident in Additional file 1: Figure S7 plate A (patient purchase), which is denser than Additional file 1: Figure S7-B (nurses’ documentation). There were many cases where patients purchased the drug with no nurse documentation, i.e. there is a more drug purchase than nurse intake documentation. For example, patient # 60 purchased the drug on seven months during our study period, but only on one of these months did the nurse document the patient was using the medication.

Additional file 1: Figure S7-D (adherence) shows an overall calculated adherence rate for furosemide of 59.9% (Table 2), yet adherence calculation does not fully account for compliance with doctors’ orders, since it does not consider over-use patterns of the drug. This figure shows frequent purchase of the drug at quantities higher (tan) or lower (light blue) than reported by the nurse. Patient #21 purchased more than what was reported since April 2008 until the end of follow-up in all but one month. The over-adherence rate we present in this report, in the case of furosemide, does not embody this medication’s use pattern.

Often these discrepancies in dosing persist over long periods. For example, patient # 33 purchased a lower quantity of furosemide than reported by the nurse for the first and last thirds of the study period, and a higher dose for the second third of the study period. Only on two months during this time, did the nurse report match the actual patient purchase (green). Nevertheless, due to our dichotomous definition of adherence, patient # 33 is considered highly adherent (91.6%).

### Non-adherence to hypoglycemic agents and antidepressants

We found an unexpected low (44.1%) adherence rate to oral hypoglycemics (Table 3, Additional file 1: Figure S8). Several patients were non-adherent for long consecutive periods while only a few patients were mostly adherent. Antidepressants also had a low adherence rate (Table 2). However, due to the low patient number this calculation is prone to inaccuracies. Interesting patterns of non-adherence can be derived from the medication plates (Additional file 1: Figure S9). In several cases the medication had not been purchased for long periods (patient # 62), while the nurse did report medication use.

## Discussion

Our study in outpatient HD units demonstrates flaws in the process of nurse drug documentation and patient drug adherence, and a high degree of physician order turbulence. Our findings support previous reports of high rates of non-adherence to medication intake among HD patients, ranging from 15% to 74% [5, 7, 8, 16]. This report’s novelty stems from the presentation of the hectic drug purchase patterns for a variety of medication groups. Though patient needs and circumstances often limit complete alignment with treatment guidelines, the demonstrated pandemonium is not the result of clinical reasoning and compromise, but rather a system failure which allows chaos rather than promoting patient-staff reconciliation. Thus, the overall consistent efficacy of pharmacotherapy in the care of our HD patients may be questionable.

Multiple methods have been studied to measure adherence in the research settings, but no single measure is considered a gold standard [10, 11, 17, 18]. Although medication regimen adherence may be described as dichotomous, there are several methods of measuring adherence [17]. Assessing adherence in the daily clinical setting is challenging more than in clinical studies given less allocated resources, variable objective standards, frequent dosing modifications and medication provision in long term residence or nursing facilities [10]. In this context, the innovative methodology we applied provides a repository of patient and nurse behaviors that is discordant to previous reports claiming >95% reliability of monthly medication reviews and record validation [19]. Manley and colleagues reported 215 drug interviews in 63 patients over five months totaling 2,709 drugs reviewed in an outpatient HD clinic. They found that there were less than 5% discrepancies between the electronic medical record and patient-provided information. However, the patient-reported information had been first verified by either the nursing or the medical staff and the low discrepancy rate probably derived from differences between these verifications and the medical file registers [19].

There are several reasons why HD patients may be at particular risk for non-adherence. First, rates of adherence among patients with any chronic disease have been shown to diminish over time, coupled with the progressive complexity of treating HD patients [5, 16]. Additionally, many of the medications prescribed for dialysis patients have no immediate discernible effects, and past research has shown that adherence is likely to be highest for symptom-relieving medications [16].

Our results corroborate this conclusion: nitrates, anti-arrhythmic agents and doxazosin manifested high adherence rates (Table 2) and fewer discrepancies between nurse reporting and patient purchase (Additional file 1: Figures S4-S6). As previously shown in the general population, anti-arrhythmic agents relieve palpitations and prevent emergency room visits due to arrhythmia [20], nitrates alleviate chest pain caused by ischemic heart disease [21] and doxazosin is effective in reducing symptoms of prostate hypertrophy as well as reducing blood pressure [22]. In contrast, adherence to other medications such as statins or ACE inhibitors may not have an immediate beneficial effect on patients’ symptoms.

Since the medications in our cohort were similar to those reported in previous HD cohorts, [23] our findings of the systematic disengagement between the nurses' documentation and actual patient purchase is fascinating. Specifically, the discrepancies included either different doses of medications or documentations of drugs not purchased at all, or vice versa, drug purchase without nurse documentation. In some cases these patterns persisted over long periods. Our results could be explained by inadequate patient-nurse communication due to unwillingness to admit non-adherence, forgetfulness of patients, or lack of systematic data collection by the dialysis staff.

Under reporting of drug use also may result from unreported over-adherence, e.g. to furosemide. Additional file 1: Figure S7 provides detailed information showing frequent furosemide overuse while descriptive statistics result in a mean adherence rate of 59.9%. It is therefore quite possible that patients are making decisions regarding dosing and usage of furosemide without consulting the medical staff. Use of furosemide may also alleviate symptoms of inter-dialytic weight gain yet it is baffling that HD patients may obtain prescriptions for furosemide in addition to prescriptions by their primary nephrologist.

Taken together, the chaotic patterns of patient behavior and nurse documentation appear to result in physician ignorance of the true nature of drug intake, thereby jeopardizing the process of medical decision making and reasoning. A system failure in data transfer is apparent and regimen management is misguided. We believe it is the responsibility of the attending nephrologists to simplify the medication regimen and reduce medication burden. At the physician’s discretion, the following strategies may reduce the pill burden in HD patients:

a. stratification of the medications according to their relative significance for the individual patient; b. initiation of drugs that have not been shown to improve outcome in HD patients, such as statins, aspirin, warfarin and a number of vitamins, may be reconsidered; c. for medications with a long half-life, adherence can be improved by in-house supervised provision of the drugs thrice-weekly; d. reducing the dose turbulence of specific medications, such as phosphate binders, to make drug dosing easier to remember and adhere to. e. because multiple care providers prescribe many different drugs with potential interactions, standard and connected electronic medical records could be beneficial in monitoring the dynamics of drug therapy; f. because drug non-adherence and registration discrepancy are hardly observed for drugs administered parenterally within the HD unit [19], medications intake could be supervised by the nursing staff during HD sessions.

The patterns of adherence and lack of accurate documentation described in this work are likely not unique to our HD population. Lack of communication, financial barriers, polypharmacy and inadequate medication reconciliation have been recognized previously as a universal obstacle to compliance with medical therapy [3, 24]. It is pertinent to establish an honest and non-judgmental relationship with both patients and dialysis care givers to better the credibility of medication use in dialysis patients.

### Limitations of the study

One limitation to our study is the small sample of patients and the failure to locate all medical files for almost a third (31.8%) of the HD patients who were selected randomly for the study. Nevertheless, the characteristics of our analyzed group are similar to previous studies [19, 25].

Another limitation may have derived from incomplete computerized pharmacy dispensing caused by purchasing drugs at a pharmacy not affiliated with CHS. However, these events are rare because in our healthcare system HD patients receive most drugs either free of charge or at a negligible cost at pharmacies affiliated with CHS.

During data analysis, measurement of adherence was possibly skewed towards overestimation of adherence. First, we corrected for excess medication purchase. Second, months preceded by no nurse documentation, were omitted from calculation in order to avoid non-adherence designation due to ignorance of doctors’ orders. However, these possible biases do not undermine, and in fact reinforce our findings regarding the perplexing patterns of drug non-adherence in nurse documentation and physician orders within chronic HD ambulatory units.

## Conclusions

Non-adherence to medication regimens in HD patients appears to be diverse as well as extensive. Drug adherence varies among drug groups, among patients, and over time. The patterns of drug purchase and nurse reports resonate on a frequency that descriptive means could not tune to. Furthermore, given the above results, physician decision-making are often misguided and may be flawed. To address these issues, communications between HD unit staff and patients, between HD unit staff and other doctors, and possibly between pharmacies’ datasets and the HD units, should be encouraged. Patient empowerment and shared responsibilities should be explored as avenues to restore accuracy in reports and prescription. Nephrologists may need to acknowledge that HD patient non-adherence is an important factor in their decision-making process and that reduction of patients’ pill burden may paradoxically improve their medical care.

## Electronic supplementary material

Additional file 1: **Figures and plates structured similarly to Figure** 1 **(plate a – patient purchase; plate b – Nurse documentation; plate c – adherence; plate d – comparing patient purchase and nurse documentation).** **Figure S1.** color legend. **Figure S2.** beta blockers. **Figure S3.** statins. **Figure S4.** anti-arrhythmics. **Figure S5.** nitrates. **Figure S6.** doxazocin. **Figure S7.** furosemide. **Figure S8.** oral hypoglycemic. **Figure S9.** anti depressants. **Figure S10.** calcium carbonate. **Figure S11.** alfacalcidiol. **Figure S12.** aspirin. **Figure S13.** calcium channel blockers. (PPTX 1 MB)
