# Supplementary material for: Summary adherence estimates do not portray the true incongruity between drug intake, nurse documentation and physicians’ orders
Source: BMC Nephrol. 2014 Oct 23;15:170. doi: 10.1186/1471-2369-15-170 (PMC4230519; doi:10.1186/1471-2369-15-170)
Supplement: Supplementary file 1 — Additional file 1: Figures and plates structured similarly to Figure 1 (plate a – patient purchase; plate b – Nurse documentation; plate c – adherence; plate d – comparing patient purchase and nurse documentation). Figure S1. color legend. Figure S2. beta blockers. Figure S3. statins. Figure S4. anti-arrhythmics. Figure S5. nitrates. Figure S6. doxazocin. Figure S7. furosemide. Figure S8. oral hypoglycemic. Figure S9. anti depressants. Figure S10. calcium carbonate. Figure S11. alfacalcidiol. Figure S12. aspirin. Figure S13. calcium channel blockers. (PPTX 1 MB) [file 12882_2014_860_MOESM1_ESM.pptx]

## Slide 1
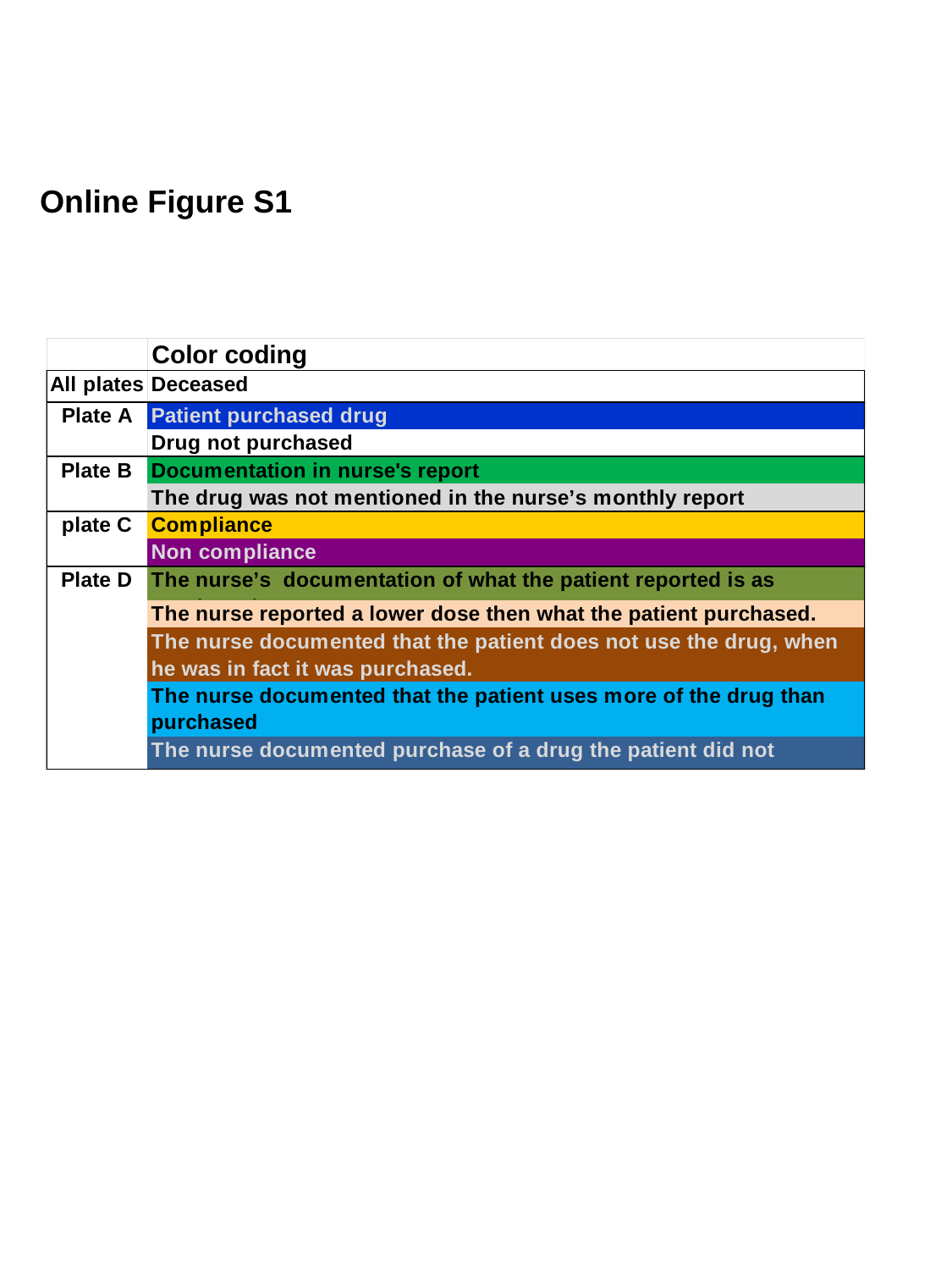

Online Figure S1

## Slide 2
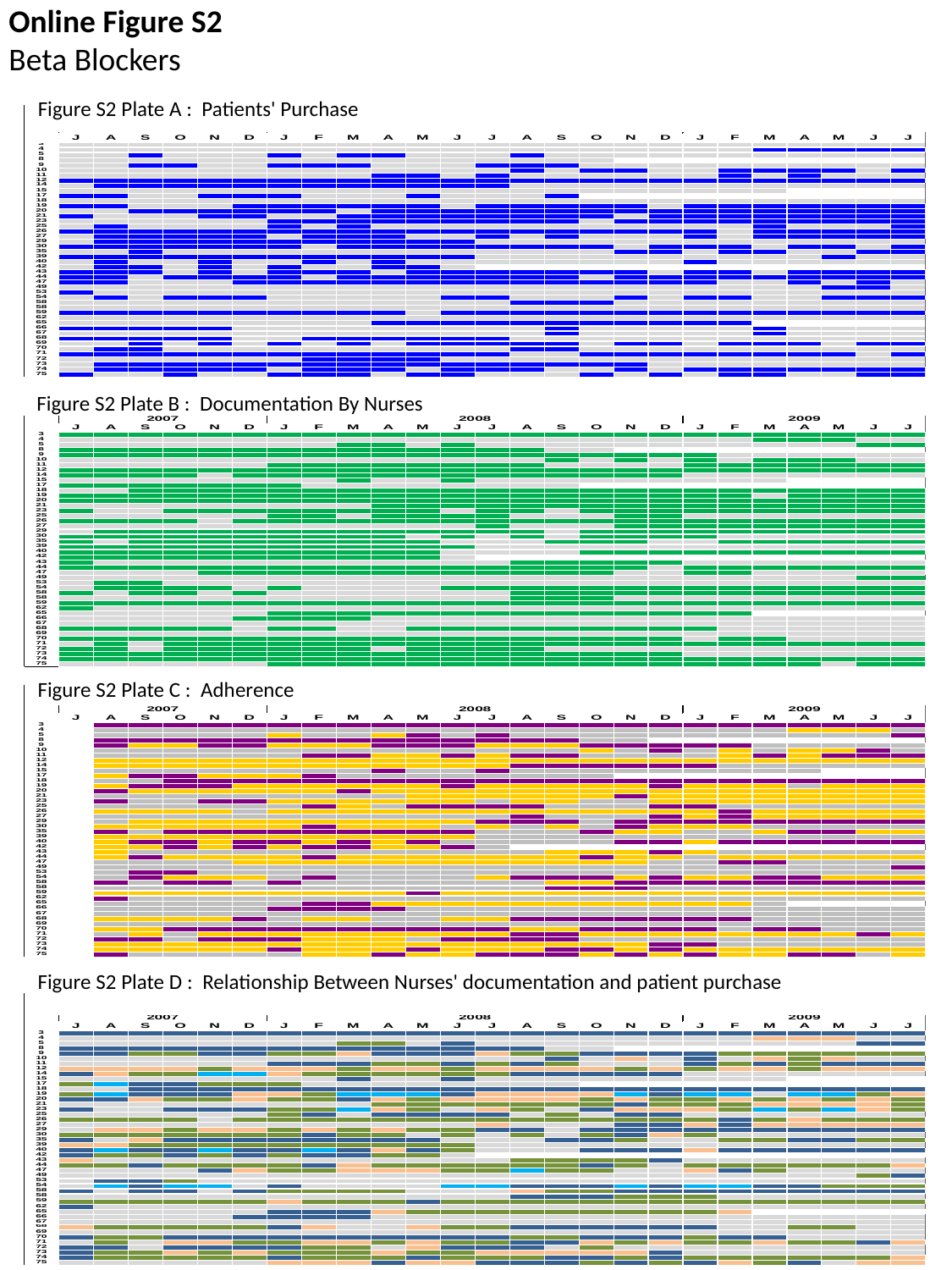

Online Figure S2
Beta Blockers
Figure S2 Plate A : Patients' Purchase
Figure S2 Plate B : Documentation By Nurses
Figure S2 Plate C : Adherence
Figure S2 Plate D : Relationship Between Nurses' documentation and patient purchase

## Slide 3
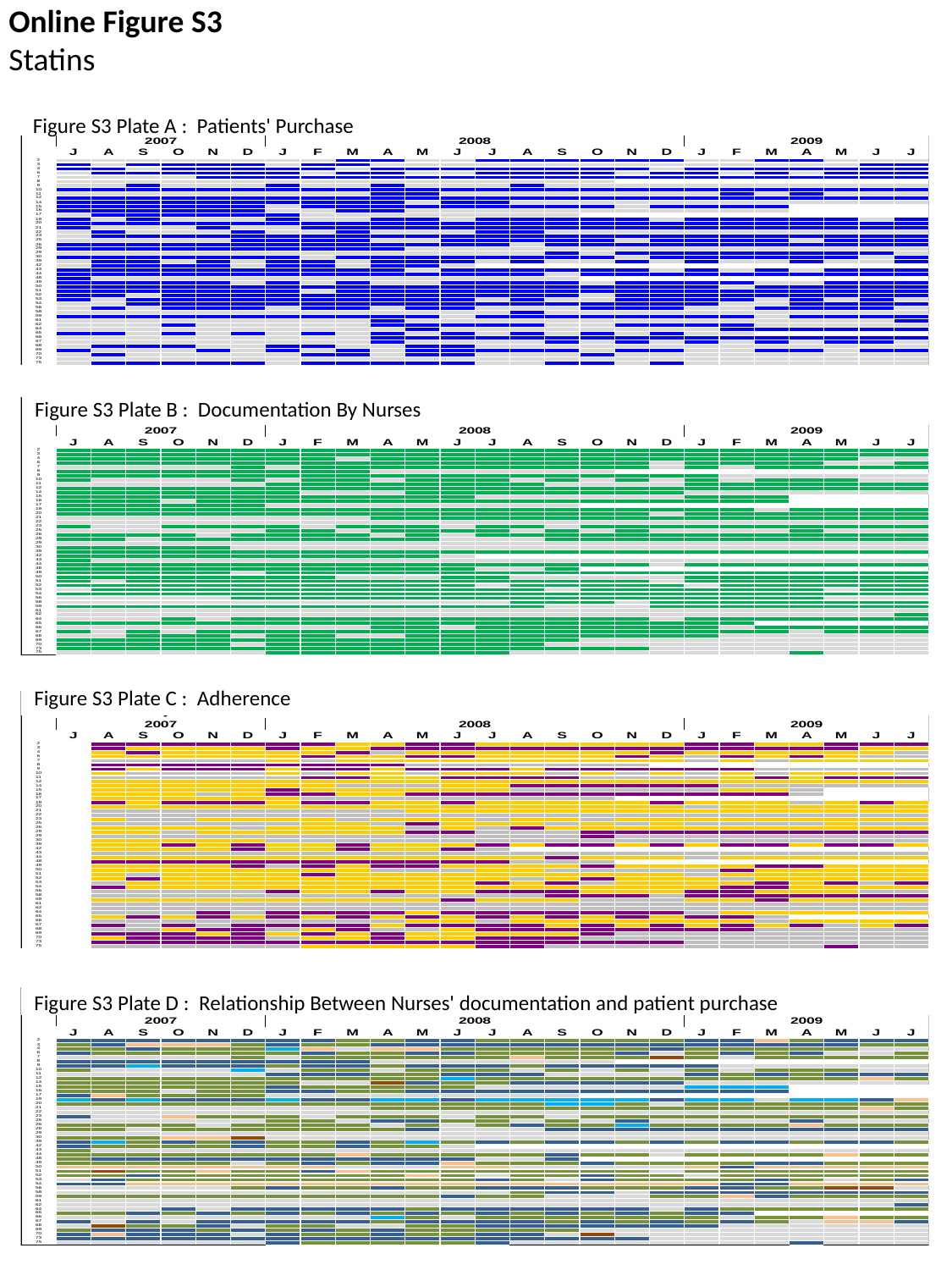

Online Figure S3
Statins
Figure S3 Plate A : Patients' Purchase
Figure S3 Plate B : Documentation By Nurses
Figure S3 Plate C : Adherence
Figure S3 Plate D : Relationship Between Nurses' documentation and patient purchase

## Slide 4
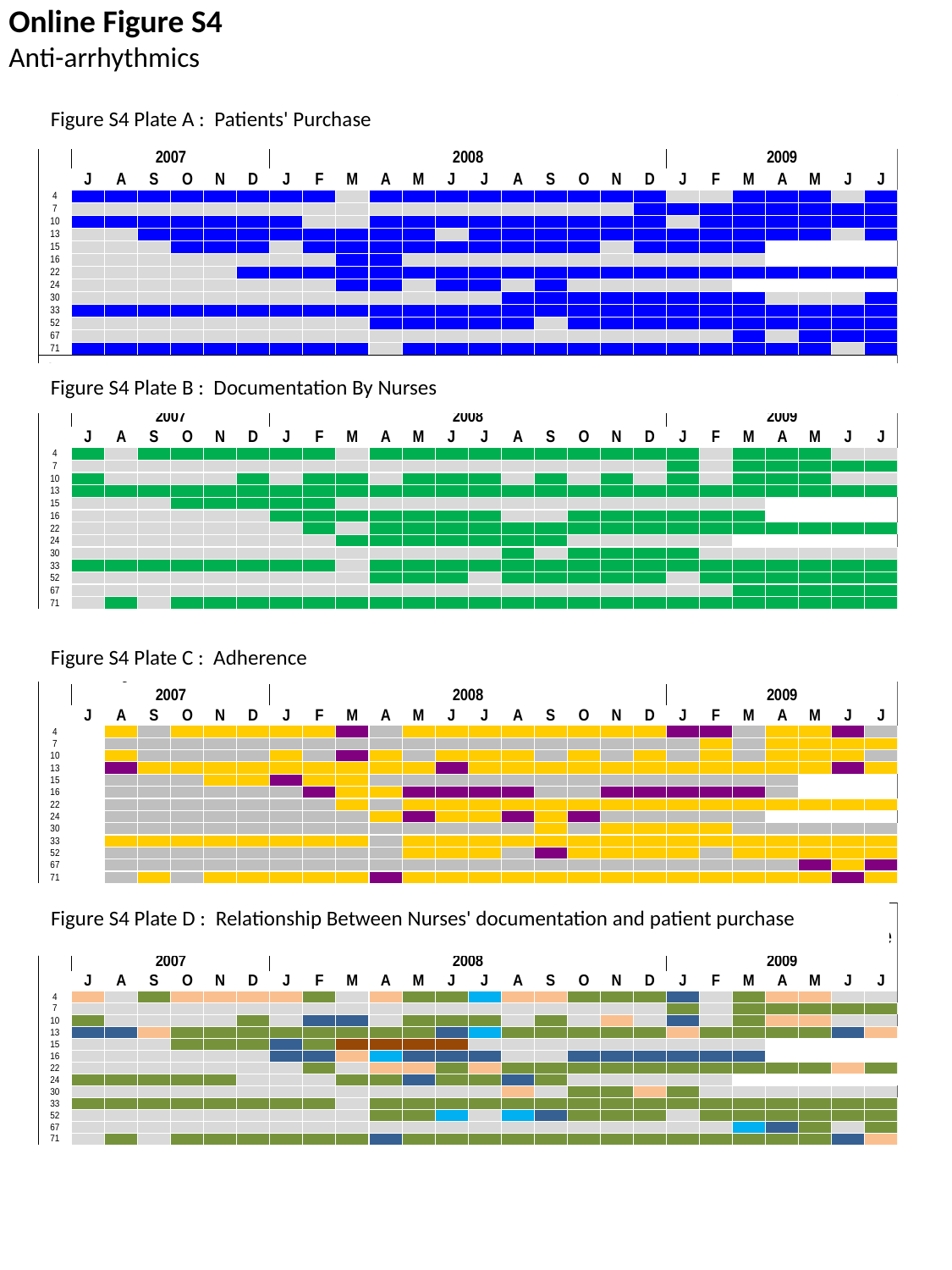

Online Figure S4
Anti-arrhythmics
Figure S4 Plate A : Patients' Purchase
Figure S4 Plate B : Documentation By Nurses
Figure S4 Plate C : Adherence
Figure S4 Plate D : Relationship Between Nurses' documentation and patient purchase

## Slide 5
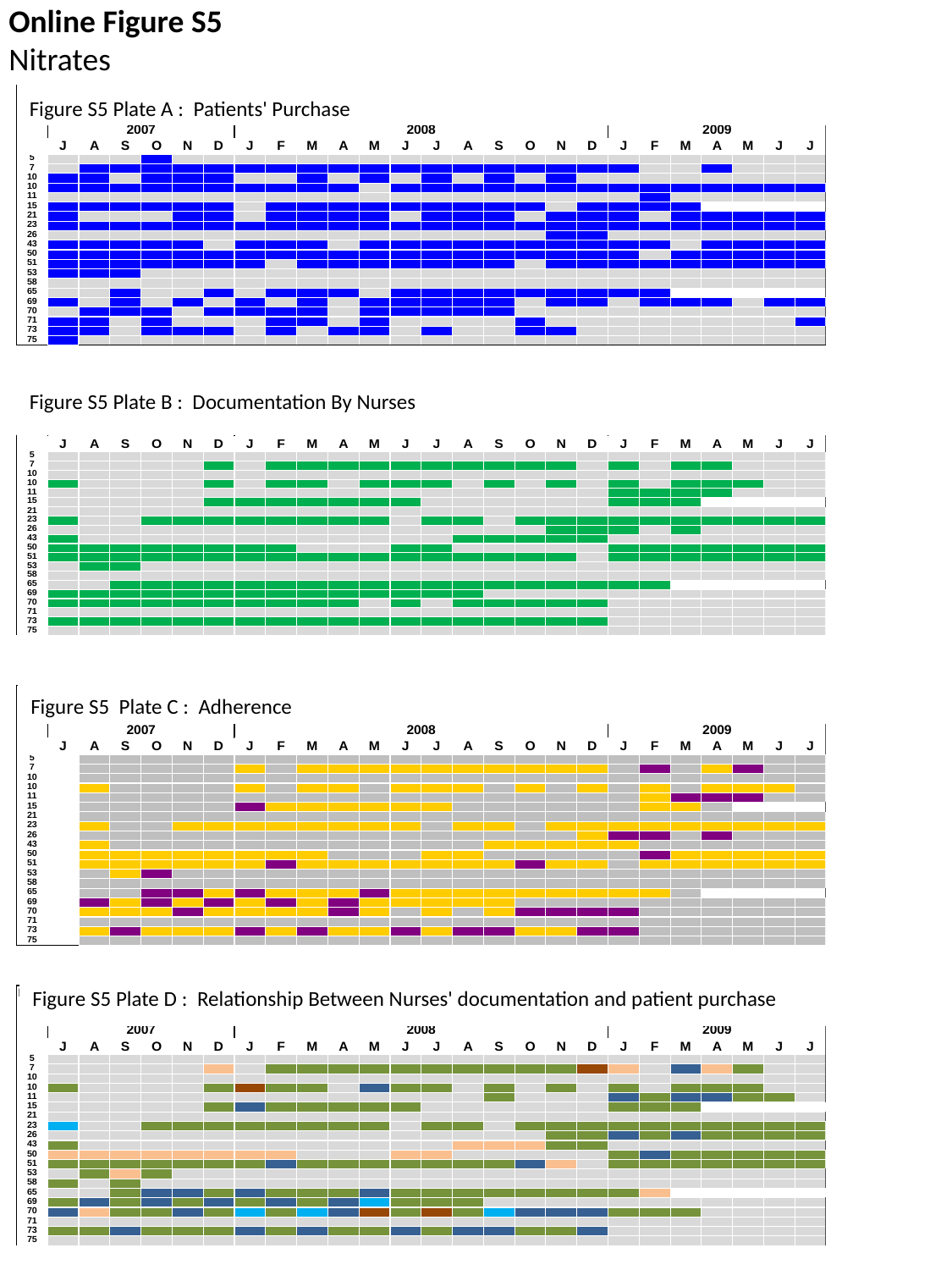

Online Figure S5
Nitrates
Figure S5 Plate A : Patients' Purchase
Figure S5 Plate B : Documentation By Nurses
Figure S5 Plate C : Adherence
Figure S5 Plate D : Relationship Between Nurses' documentation and patient purchase

## Slide 6
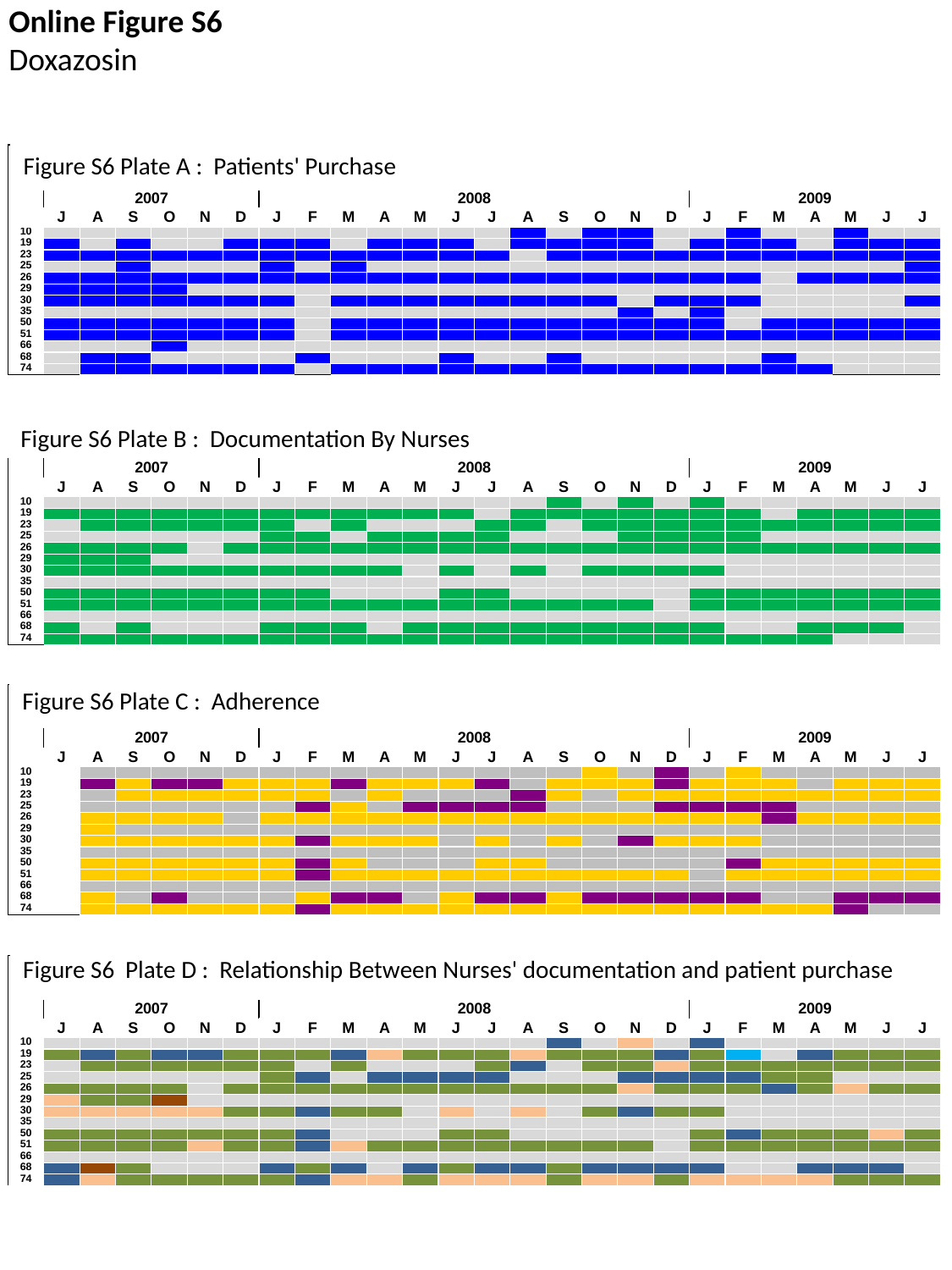

Online Figure S6
Doxazosin
Figure S6 Plate A : Patients' Purchase
Figure S6 Plate B : Documentation By Nurses
Figure S6 Plate C : Adherence
Figure S6 Plate D : Relationship Between Nurses' documentation and patient purchase

## Slide 7
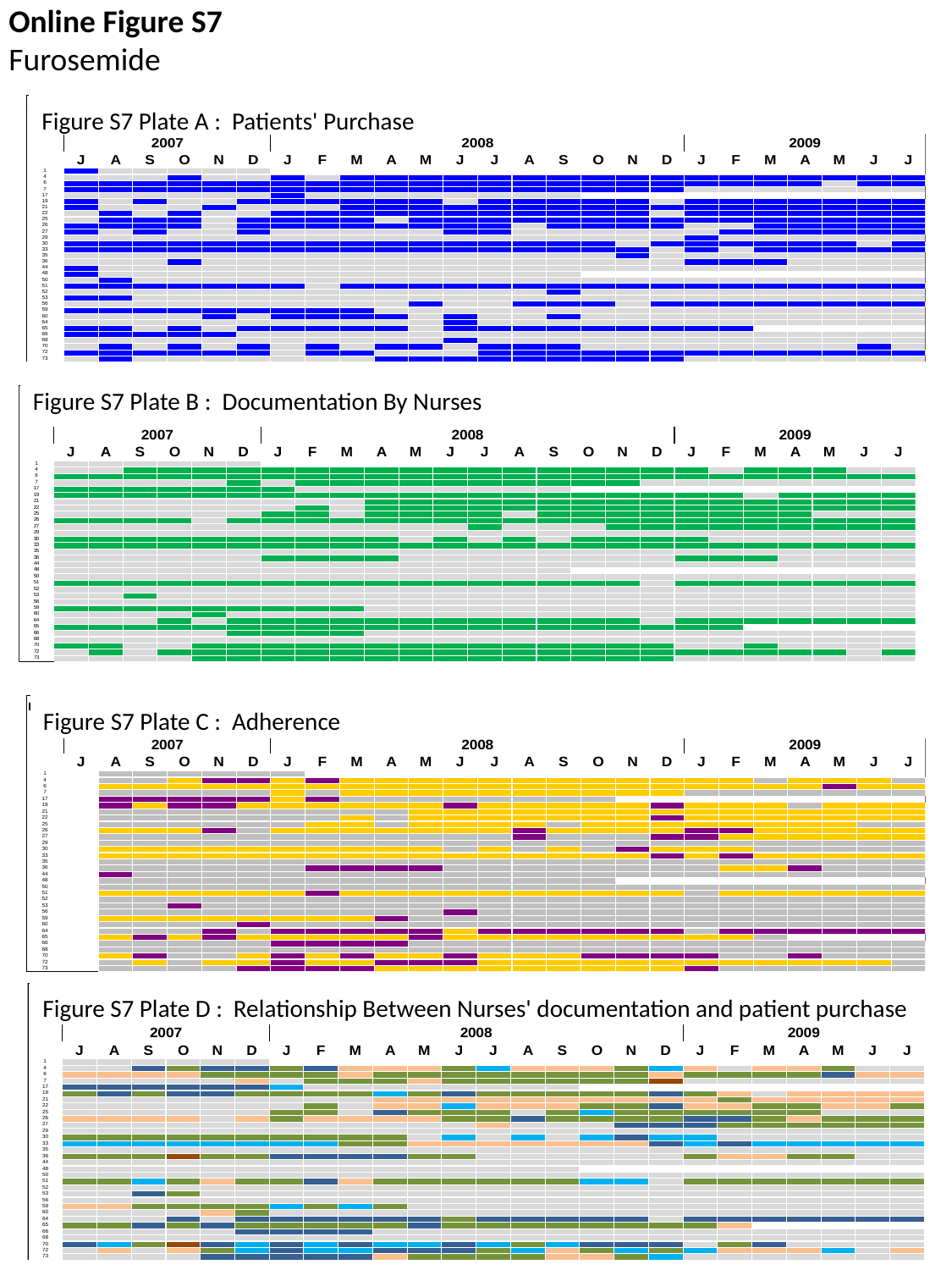

Online Figure S7
Furosemide
Figure S7 Plate A : Patients' Purchase
Figure S7 Plate B : Documentation By Nurses
Figure S7 Plate C : Adherence
Figure S7 Plate D : Relationship Between Nurses' documentation and patient purchase

## Slide 8
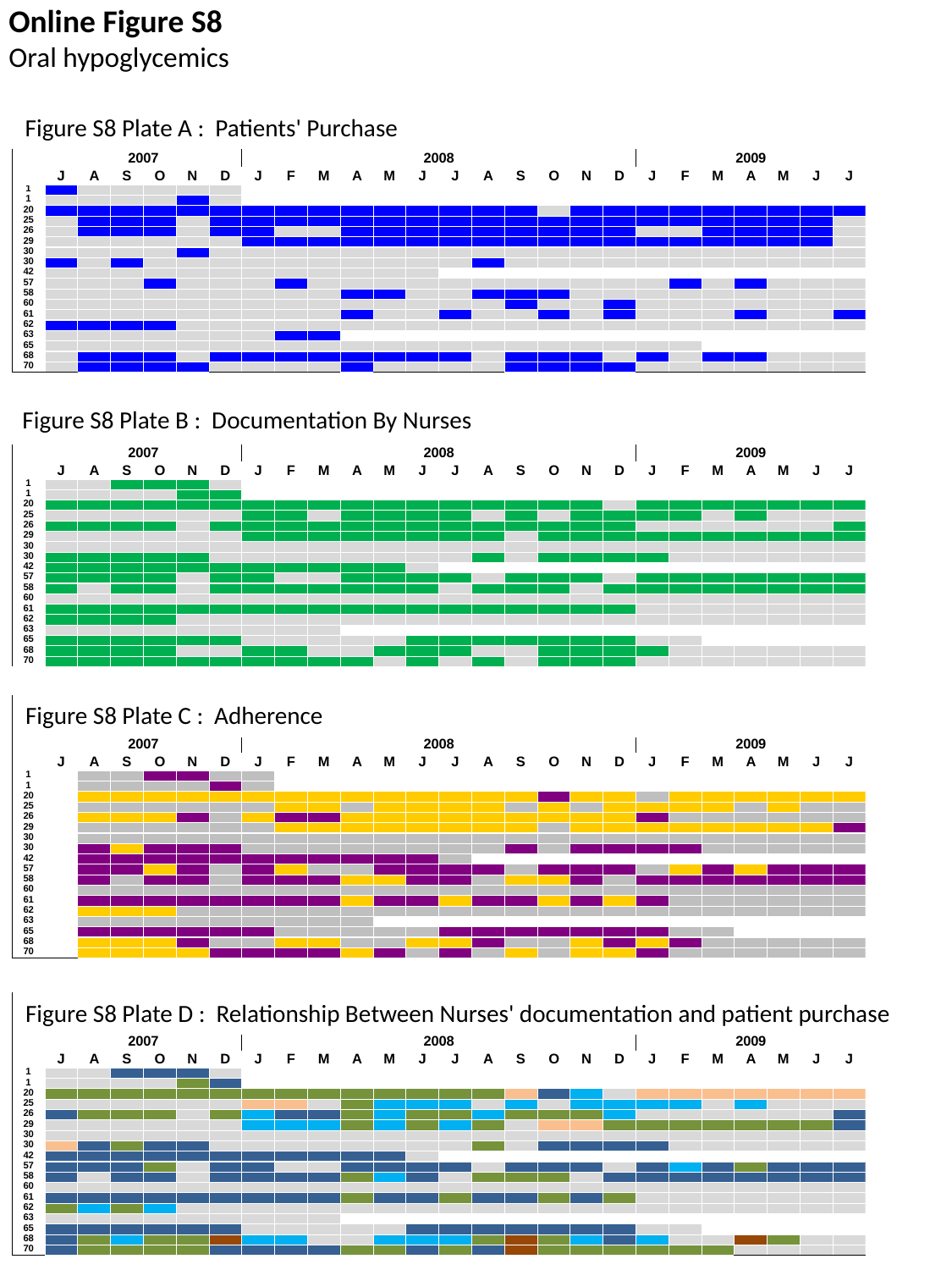

Online Figure S8
Oral hypoglycemics
Figure S8 Plate A : Patients' Purchase
Figure S8 Plate B : Documentation By Nurses
Figure S8 Plate C : Adherence
Figure S8 Plate D : Relationship Between Nurses' documentation and patient purchase

## Slide 9
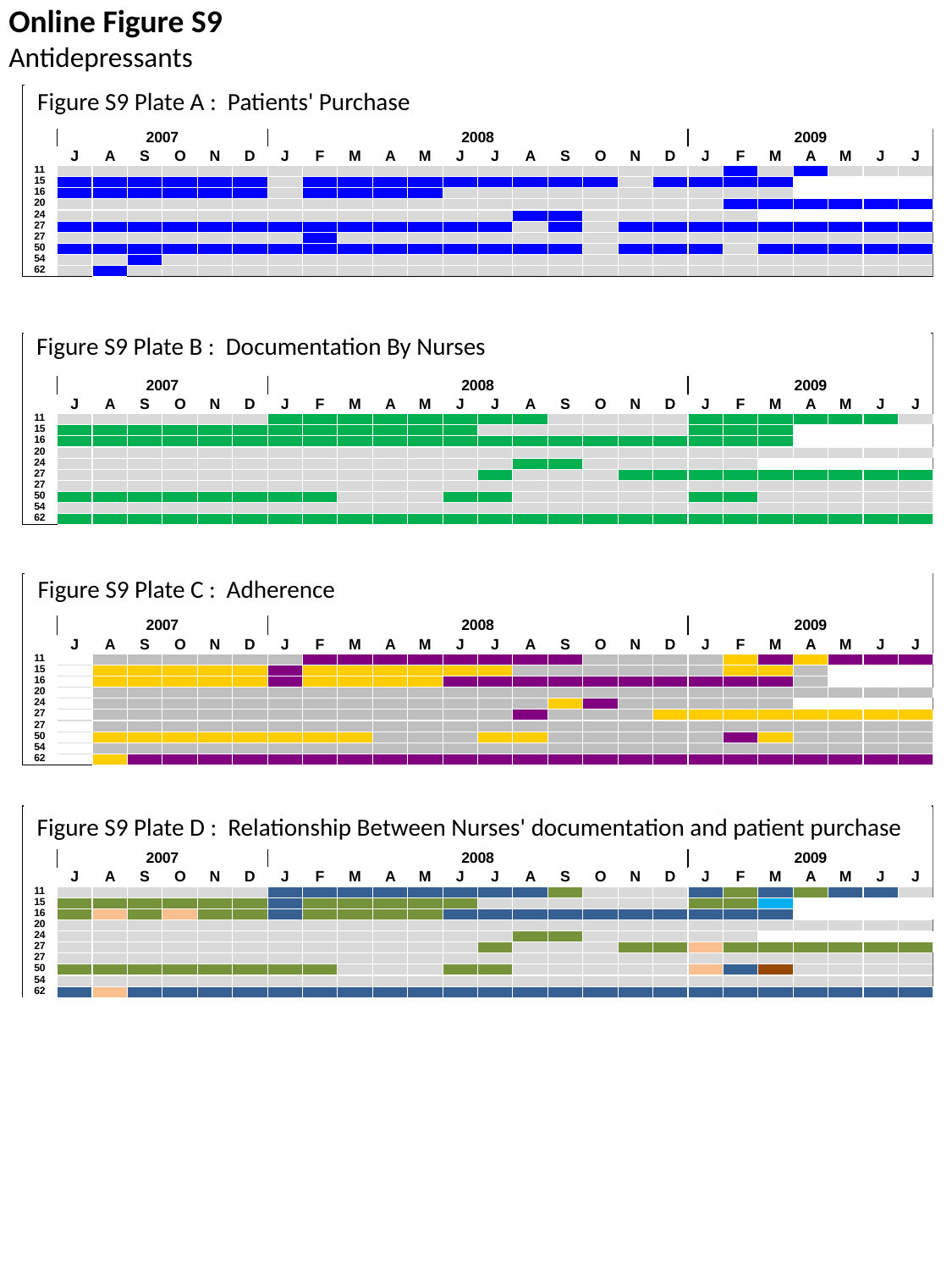

Online Figure S9
Antidepressants
Figure S9 Plate A : Patients' Purchase
Figure S9 Plate B : Documentation By Nurses
Figure S9 Plate C : Adherence
Figure S9 Plate D : Relationship Between Nurses' documentation and patient purchase

## Slide 10
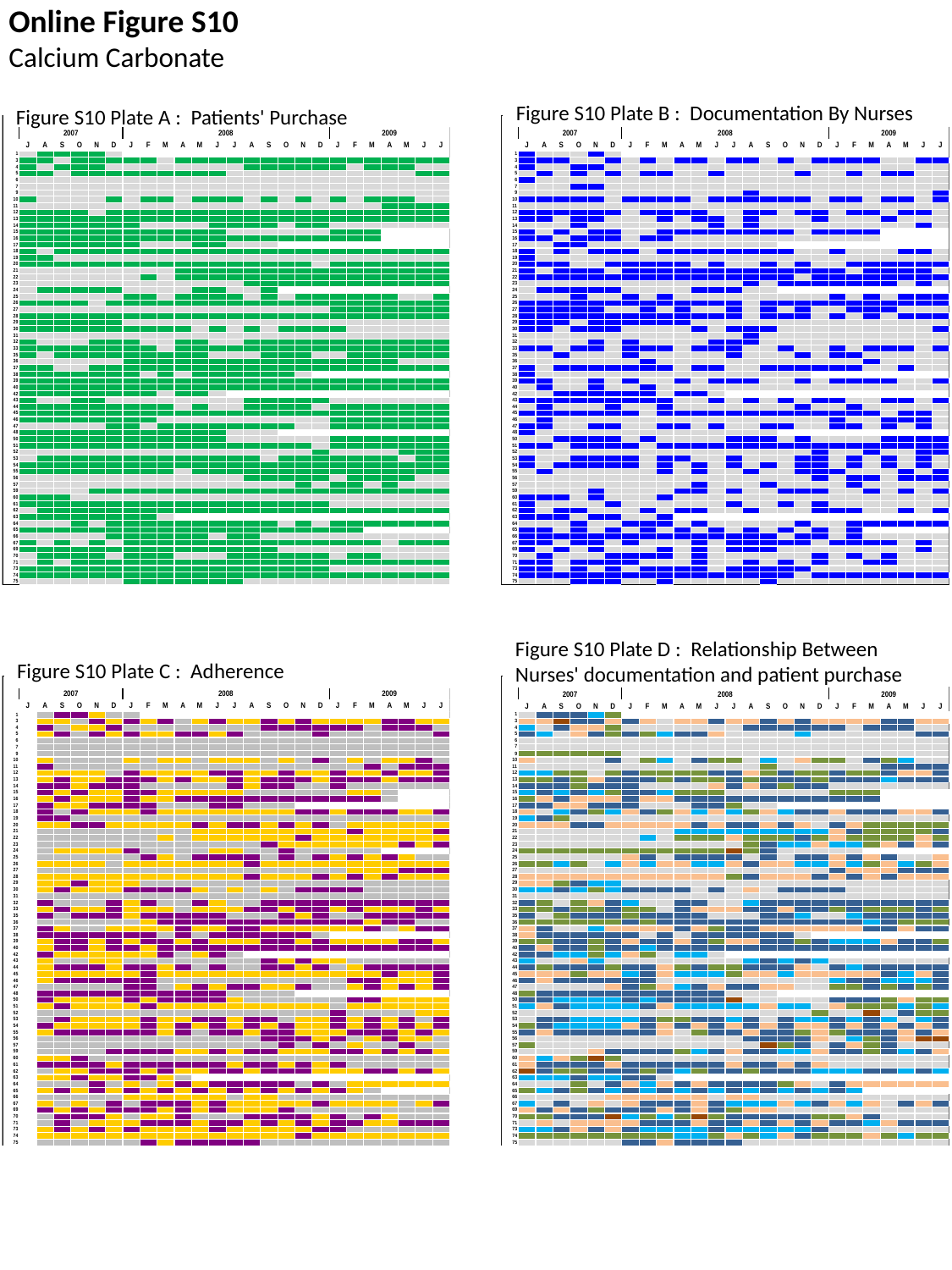

Online Figure S10
Calcium Carbonate
Figure S10 Plate B : Documentation By Nurses
Figure S10 Plate A : Patients' Purchase
Figure S10 Plate D : Relationship Between Nurses' documentation and patient purchase
Figure S10 Plate C : Adherence

## Slide 11
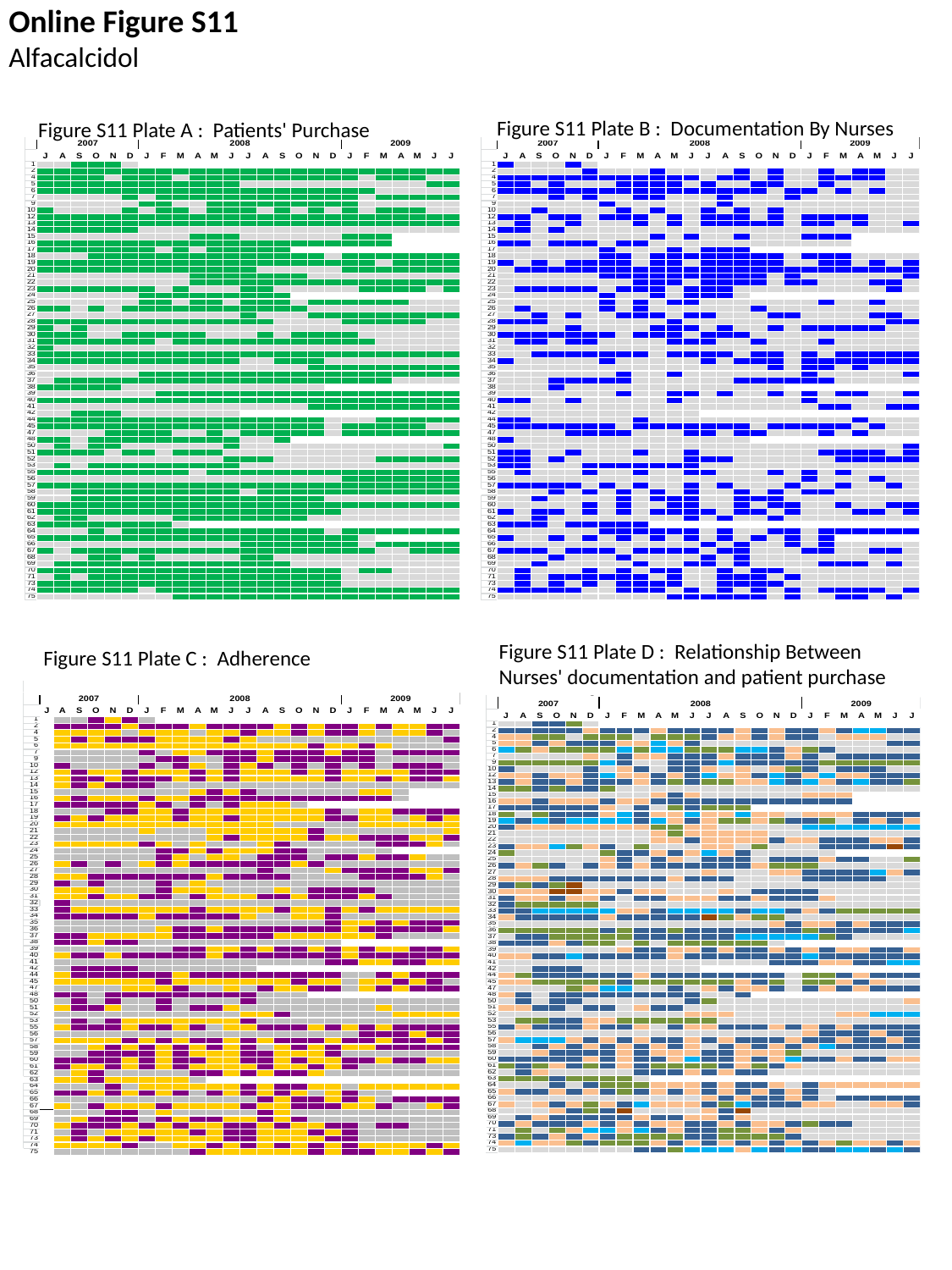

Online Figure S11
Alfacalcidol
Figure S11 Plate B : Documentation By Nurses
Figure S11 Plate A : Patients' Purchase
Figure S11 Plate D : Relationship Between Nurses' documentation and patient purchase
Figure S11 Plate C : Adherence

## Slide 12
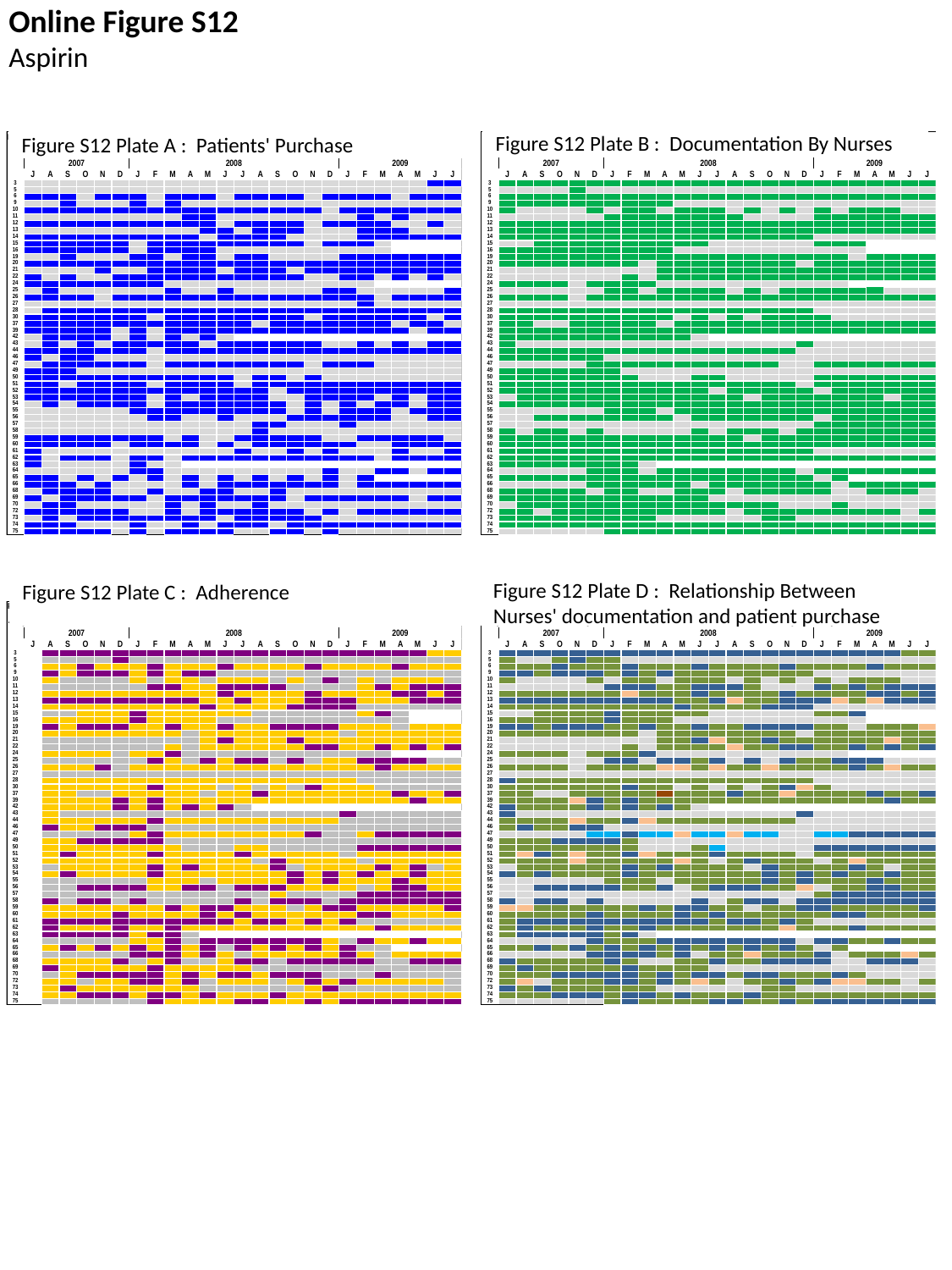

Online Figure S12
Aspirin
Figure S12 Plate B : Documentation By Nurses
Figure S12 Plate A : Patients' Purchase
Figure S12 Plate C : Adherence
Figure S12 Plate D : Relationship Between Nurses' documentation and patient purchase

## Slide 13
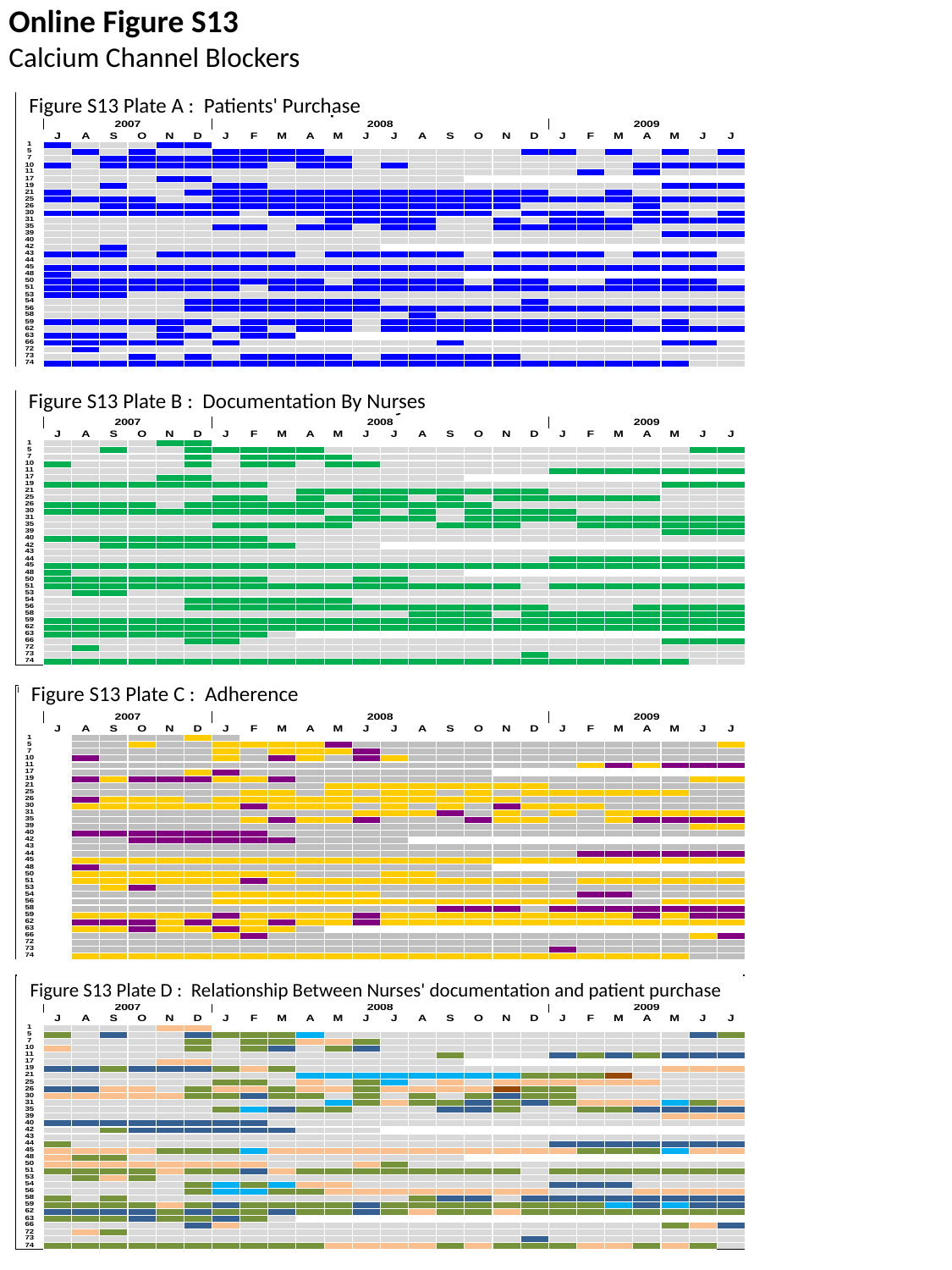

Online Figure S13
Calcium Channel Blockers
Figure S13 Plate A : Patients' Purchase
Figure S13 Plate B : Documentation By Nurses
Figure S13 Plate C : Adherence
Figure S13 Plate D : Relationship Between Nurses' documentation and patient purchase
